# An anthocyanin-rich extract from *Zea mays* L. *var. ceratina* alleviates neuronal cell death caused by hydrogen peroxide-induced cytotoxicity in SH-SY5Y cells

**DOI:** 10.1186/s12906-024-04458-6

**Published:** 2024-04-17

**Authors:** Nootchanat Mairuae, Nut Palachai, Parinya Noisa

**Affiliations:** 1 Faculty of Medicine, Mahasarakham University, Mahasarakham, 44000 Thailand; 2 School of Biotechnology, Institute of Agricultural Technology, Suranaree University of Technology, Nakhon Ratchasima, 30000 Thailand

**Keywords:** Dementia, Neuronal cell death, Oxidative stress, Apoptosis, Anthocyanin-rich extract, *Zea mays* L. *var. ceratina*, Purple waxy corn cob, Neuroprotective effects

## Abstract

The incidence of dementia is rising, with neuronal cell death from oxidative stress and apoptosis recognized as a significant contributor to its development. However, effective strategies to combat this condition are lacking, necessitating further investigation. This study aimed to assess the potential of an anthocyanin-rich extract from *Zea mays* L. *var. ceratina* (AZC) in alleviating neuronal cell death.

Neurotoxicity was induced in SH-SY5Y cells using hydrogen peroxide (H_2_O_2_) at a concentration of 200 µM. Cells were pretreated with varying doses (31.25 and 62.5 µg/mL) of AZC. Cell viability was assessed using the MTT assay, and molecular mechanisms including reactive oxygen species (ROS) levels, antioxidant enzyme activities (catalase (CAT), superoxide dismutase (SOD), and glutathione peroxidase (GSH-Px)), malondialdehyde (MDA) levels for oxidative stress, and the activation of extracellular signal-regulated kinases 1 and 2 (ERK1/2), cAMP response element-binding protein (CREB), and apoptotic factors (B-cell lymphoma 2 (Bcl-2), caspase 3) were explored.

Results showed that AZC significantly improved cell viability, reduced ROS production and MDA levels, and downregulated caspase 3 expression. It enhanced CAT, SOD, and GSH-Px activities, activated ERK1/2 and CREB, and upregulated Bcl-2 expression. These findings support the neuroprotective effects of AZC, suggesting it activates ERK1/2, leading to CREB activation and subsequent upregulation of Bcl-2 expression while suppressing caspase 3. AZC may mitigate neuronal cell death by reducing ROS levels through enhanced scavenging enzyme activities.

In conclusion, this study underscores the potential of AZC as a neuroprotective agent against neuronal cell death. However, further investigations including toxicity assessments, in vivo studies, and clinical trials are necessary to validate its benefits in neuroprotection.

**Supplementary Information:**

The online version contains supplementary material available at 10.1186/s12906-024-04458-6.

## Introduction

Dementia, a broad term used to describe a group of cognitive disorders characterized by memory decline, impaired thinking, behavioral changes, and reduced ability to perform daily activities, is a significant global health concern [1]. Its prevalence is increasing, exerting a substantial impact on patients, families, caregivers, and societies worldwide [2]. The underlying mechanisms of dementia are complex, involving various factors such as oxidative stress, inflammation, mitochondrial dysfunction, and neuronal damage [3, 4].

Oxidative stress, characterized by an imbalance between the generation of reactive oxygen species (ROS) and the body’s antioxidant defenses, plays a central role in dementia’s pathogenesis [5]. The brain’s heightened metabolic rate, coupled with its lipid-rich composition and limited antioxidant capacity, renders neurons especially susceptible to oxidative harm. This imbalance leads to lipid peroxidation, protein oxidation, and DNA damage within neurons, culminating in cellular dysfunction and neuronal death [6].

Simultaneously, chronic neuroinflammation exacerbates the neurodegenerative process seen in dementia [7]. Activated microglia, the brain’s resident immune cells, release pro-inflammatory cytokines and other mediators in response to pathological stimuli. These molecules further damage neurons, disrupt synaptic connections, and perpetuate neurodegeneration, hastening cognitive decline [8].

Mitochondrial dysfunction further compounds the pathological landscape of dementia, resulting in impaired energy production, ROS overproduction, and compromised cellular resilience [9]. Mitochondria, crucial for ATP synthesis, malfunction under pathological conditions, compromising cellular energy production and rendering neurons vulnerable to oxidative stress and apoptosis. Consequently, mitochondrial dysfunction not only contributes to oxidative stress but also accelerates neuronal death, exacerbating dementia’s progression [10].

Moreover, the cumulative impact of oxidative stress, inflammation, and mitochondrial dysfunction compromises neuronal integrity, leading to widespread neuronal damage and synaptic dysfunction [11]. Neurons, critical for cognitive function, succumb to pathological insults, resulting in synaptic loss, dendritic atrophy, and eventual neuronal death. As neuronal networks deteriorate, cognitive decline ensues, underscoring the profound repercussions of neurodegeneration on cognitive function in dementia [12].

Current treatment strategies primarily focus on symptom management, disease progression deceleration, and improvement of individuals’ quality of life [13]. Unfortunately, targeted treatments are presently unavailable, and the effectiveness of existing therapies remains limited. Consequently, there is an urgent need to explore preventive approaches.

In recent years, there has been a growing interest in the potential health benefits of anthocyanins, a group of natural pigments belonging to the flavonoid family. Anthocyanins are commonly found in various parts of plants. They have attracted attention for their potential role in brain health and their ability to prevent or manage dementia [14, 15]. Several preclinical studies explored the protective effects of anthocyanins against dementia and cognitive decline. These studies revealed that anthocyanins possess a wide range of beneficial properties, including antioxidant, anti-inflammatory, and anti-apoptotic effects [16–18]. These findings suggest that anthocyanins may possess neuroprotective properties and could contribute to the prevention or delay of cognitive impairment. As a result, the neuroprotective potential of anthocyanins against hydrogen peroxide-induced cytotoxicity has gained significant attention.

*Zea mays* L. *var. ceratina* is a variety of corn characterized by its purple-colored husk and kernels. It is highly regarded for its abundant anthocyanin content, which makes it a valuable source of bioactive compounds, particularly those belonging to the anthocyanin group. Numerous studies have demonstrated that *Zea mays* L. *var. ceratina* extract contains a diverse array of anthocyanins, including derivatives of cyanidin, peonidin, and pelargonidin [19]. These anthocyanins have been extensively investigated for their potential health benefits and medicinal properties [20, 21].

Based on the aforementioned information regarding the positive modulation effects of the anthocyanin-rich extract from *Zea mays* L. *var. ceratina* (AZC) against neuronal cell death, it has been hypothesized that the extract should possess biological activities associated with the pathophysiology of dementia and may enhance neuronal cell survival in the presence of hydrogen peroxide-induced cytotoxicity in SH-SY5Y cells. To investigate the potential underlying mechanisms, various parameters were evaluated, including the activity of scavenging enzymes such as CAT, SOD, and GSH-Px, levels of MDA and ROS production, as well as the expression levels of ERK1/2, CREB, and apoptotic factors, including Bcl-2 and caspase 3.

## Materials and methods

### Preparation of anthocyanin-rich extract from *Zea mays* L. *var. ceratina*

The *Zea mays* L. *var. ceratina* (voucher specimen KKU 25979) hereafter referred to as AZC utilized in this study was sourced from the Faculty of Agriculture at Khon Kaen University in Khon Kaen province, Thailand. Following authentication, the AZC underwent a meticulous cleaning and drying procedure at 60 °C for 48 h in a Memmert GmbH oven (USA). Subsequently, the dried material was finely pulverized into a powder. From this powdered material, extracts were prepared through a maceration process involving distilled water, a 50% hydro-alcoholic solution, and a 95% hydro-alcoholic solution. The resulting extract underwent centrifugation at 3,000 revolutions per minute (rpm) for 10 min and was then filtered using Whatman No. 1 filter paper. The solvent from the filtrate was removed employing a rotary evaporator and a freeze dryer from Labconco Corporation (Kansas City, MO, USA). These extracts were employed for the determination of phenolic compounds, flavonoids, anthocyanins, as well as their assessment of antioxidant and anti-inflammatory activities.

### Determination of the fingerprint chromatogram

The fingerprint chromatogram of AZC was established through high-performance liquid chromatography (HPLC) employing a Waters® system equipped with a Waters® 2998 photodiode array detector. Separation was achieved using Purospher® STAR, C-18 encapped (5µM), LiChro-CART® 250 − 4.6, and HPLC-Cartridge, Sorbet Lot No. HX255346 (Merck, Germany).

In this study, the mobile phase gradient comprised 100% methanol (solvent A) (Fisher Scientific, USA) and 2.5% acetic acid (solvent B) (Fisher Scientific, USA) in deionized (DI) water. The gradient elution was conducted at a flow rate of 1.0 mL/min, with the following gradient profile: 0–17 min, 70% A; 18–20 min, 100% A; 20.5–25 min, 10% A. Before administration, the sample underwent filtration (0.45 μm, Millipore), and a 20 µL aliquot of the sample was directly utilized. The chromatogram was assessed at 280 nm using a UV detector, and data analysis was carried out employing EmpowerTM3 [22].

### Determination of the total content of phenolic compounds, flavonoids, and anthocyanins

The total phenolic compounds content was assessed using the Folin-Ciocalteu colorimetric method with a microplate reader (iMark™ Microplate Absorbance Reader). In brief, 20 µL of the extract was mixed with a freshly prepared reagent containing 20 µL of 50% v/v Folin-Ciocalteu reagent (Sigma-Aldrich, USA) and 158 µL of distilled water. After an 8-minute incubation, 30 µL of 20% Na_2_CO_3_ (Sigma-Aldrich, USA) was added. The mixture was further incubated in a dark room at 25 °C for 2 h, and the absorbance was measured at 765 nm [23]. The results were expressed as milligrams of gallic acid equivalent (GAE) per gram of extract. A standard calibration curve was constructed using various concentrations of gallic acid (Sigma-Aldrich, USA).

The total flavonoid content in the extract was determined using the aluminum chloride method. In short, 100 µL of the extract at different concentrations were mixed with 100 µL of 2% methanolic aluminum chloride (Sigma-Aldrich, USA). The mixture was incubated at 25 °C in a dark room for 30 min, and the absorbance at 415 nm was measured against an appropriate blank [24]. The results were expressed as µg quercetin equivalent/g extract. Various concentrations of quercetin (Sigma-Aldrich, USA) were used to prepare a standard calibration curve.

The assessment of total anthocyanin content was carried out using a pH-differential method. To summarize, 1 mL of the extract was mixed with 2 mL of 0.025 M potassium chloride pH 1.0 and 2 mL of 0.4 M sodium acetate pH 4.5. The mixed solutions were incubated at 25 °C for 10 min, and then the absorbance was measured at 520 and 720 nm using a UV-spectrophotometer (Pharmacia LKB-Biochrom 4060). The anthocyanin content was calculated and expressed as mg of cyanidin-3-glucoside equivalents/g extract. This calculation utilized a molar extinction coefficient (ɛ) of cyanidin-3-O-glucoside (26,900 L mol^− 1^ cm^− 1^) and a molecular weight (MW) of 449.2 g mol^− 1^) [25].

### Determination of biological activities

#### Antioxidant activities

**DPPH** For the measurement of free radical-scavenging activity, the 1,1-diphenyl-2-picrylhydrazyl radical (DPPH) was employed. A methanolic solution of DPPH at a concentration of 0.1 mM was prepared. Then, 2 mL of this solution was mixed with 0.3 mL of various extract concentrations (ranging from 1 to 100 mg/mL) and incubated for 30 min at 25 °C. After incubation, the absorbance at 517 nm was measured against a blank (without the extract) using a microplate reader (iMark™ Microplate Absorbance Reader) [26]. The results were expressed as EC_50_, representing the concentration in µg/mL required to inhibit radical formation by 50%.

**FRAP** The ferric tripyridyltriazine (Fe^3+^-TPTZ) to ferrous tripyridyltriazine (Fe^2+^-TPTZ) conversion was utilized for the determination of ferric reducing antioxidant power (FRAP) of the extract. A freshly prepared FRAP solution containing 20 mM ferric chloride (FeCl_3_) (Sigma-Aldrich, USA), 300 mM acetate buffer (Sigma-Aldrich, USA), and 10 mM TPTZ (Sigma-Aldrich, USA) was mixed at a ratio of 1:10:1, respectively. 90 µL of the FRAP reagent was mixed with 10 µL of the extract and incubated at 37 °C for 10 min. The absorbance at 593 nm was measured against a blank [27]. The results were expressed as EC_50_ values.

**ABTS** The determination of free radical-scavenging activity using 2,2-Azinobis-3-ethylbenzothiazoline-6-sulfonic acid (ABTS) involved the preparation of an ABTS^•+^ solution by mixing 7 mM ABTS (Sigma-Aldrich, USA) and 2.45 mM potassium persulfate (K_2_S_2_O_8_) (Sigma-Aldrich, USA) at a ratio of 2:3. For assays, 30 µL of the extract at various concentrations was mixed with 120 µL of distilled water and 30 µL of ethanol. The mixture was reacted with 3 mL of ABTS^•+^ solution, and the absorbance was measured at 734 nm using a spectrophotometer (Pharmacia LKB-Biochrom 4060) [28]. The radical scavenging activity was expressed as EC_50_ value.

#### Cyclo-oxygenase-II (COX-II) inhibition

To assess the cyclo-oxygenase-II (COX-II) inhibition, a colorimetric COX-II inhibitor screening assay kit (Cayman Chemical, USA) was employed. The extract’s anti-inflammatory effect on COX-II inhibition activity was determined following the manufacturer’s protocol. A COX-II working solution was prepared by dissolving the COX-II substance in 100 mM Tris-HCl buffer with a pH of 8.0, at a ratio of 1:100. In summary, a mixture containing 150 µL of assay buffer, 10 µL of the extract, 10 µL of heme (Cayman Chemical, USA), 10 µL of COX-II working solution, 20 µL of 10 µM TMPD (N, N,N’,N’-Tetramethyl-p-phenylenediamine dihydrochloride) (Sigma, USA), and 20 µL of 100 µM arachidonic acid (Cayman Chemical, USA) was added to 96-well microliter plates. The plates were then incubated at 25 °C for 30 min. Subsequently, the absorbance at 590 nm was measured, with indomethacin used as the reference standard [29]. The results were expressed as EC_50_ value.

### Cell culture and cell viability assay

#### Cell culture

The SH-SY5Y cell line used in this study was derived from a neuroblastoma bone marrow biopsy and was of neuronal origin, exhibiting neuronal cell properties. The cell line was obtained from the American Type Culture Center (ATCC Manassas, Virginia, USA) under the catalog number CRL-2266. To maintain the SH-SY5Y cells, they were cultured in DMEM medium (Gibco, USA) supplemented with 10% FBS, 1% penicillin-streptomycin, and 1% non-essential amino acids. The cells were incubated at 37 °C in a humidified atmosphere containing 5% CO_2_ [30].

For each experiment, the cells were plated at an appropriate density. Prior to the start of the experiment, the culture medium in each well was completely removed, and fresh medium containing hydrogen peroxide, with or without AZC, was added.

#### Cell viability assay

The MTT assay was performed to evaluate the in vitro cytotoxicity of hydrogen peroxide and AZC. SH-SY5Y cells were seeded in 96-well plates at a density of 1 × 10^4^ cells/well and cultured according to the previously described conditions. The cells were treated with various concentrations of hydrogen peroxide (ranging from 50 µM to 1,000 µM) and AZC (ranging from 0 µg/mL to 1,000 µg/mL) in serum-free DMEM for a duration of 24 h. The concentration of hydrogen peroxide-induced cytotoxicity used in this study was optimized to match the concentrations utilized in our previous study [30].

To investigate the potential protective effects of AZC against hydrogen peroxide-induced neurotoxicity, the cells were pre-treated with extract for 18 h. Subsequently, the media was removed, and fresh medium containing hydrogen peroxide, with or without AZC, was added. After a total treatment duration of 24 h, the culture medium was replaced with MTT reagent obtained from Sigma, USA. The final concentration of MTT reagent was 0.5 mg/mL. The cells were then incubated at 37˚C for 1 h in a humidified atmosphere containing 5% CO_2_. Following the incubation period, the MTT reagent was aspirated, and 100 µL of dimethyl sulfoxide (DMSO) was added to dissolve the insoluble purple formazan product. The absorbance of the resulting solution was measured at 570 nm using a suitable microplate reader (BioTek Synergy H1 Multimode Reader, USA) [30].

### Determination of intracellular reactive oxygen species levels

The measurement of intracellular reactive oxygen species (ROS) levels was performed using CM-H2DCFDA, a cell-permeable fluorescent probe. SH-SY5Y cells were seeded in a 96-well plate and cultured according to the aforementioned protocol. After a 24-hour incubation, the cells were pretreated with or without AZC. Subsequently, the cells were exposed to 10 µM CM-H2DCFDA and maintained at 37 °C for 30 min in a 5% CO_2_ incubator in the darkness. Following this, the cells were washed with PBS and subjected to H_2_O_2_ treatment in serum-free medium for 24 h. Within the cells, CM-H2DCFDA underwent conversion by intracellular esterases in the presence of ROS, leading to the formation of the fluorescent compound 2’,7’-dichlorofluorescein (DCF). The fluorescence intensity of DCF, which acted as an indicator of ROS levels, was quantified using a Synergy HT Multi-Mode microplate reader (BioTek instruments, Winooski, VT) with an excitation wavelength of 488 nm and an emission wavelength of 520 nm [31].

### Determination of oxidative stress status

To assess the alterations of oxidative stress markers, the cell homogenates were prepared by homogenizing the cells with 0.1 M potassium phosphate buffer solution at pH 7.4. The homogenization was performed by diluting 10 mg of sample in 50 µL of PBS. The protein concentration in the cell homogenates was determined using a Thermo Scientific NanoDrop 2000c spectrophotometer by measuring the optical absorbance at 280 nm.

CAT activity was determined based on its ability to break down hydrogen peroxide. A total of 10 µL of the prepared sample was mixed with a solution containing 25 µL of 4 M H_2_SO_4_, 50 µL of 30 mM hydrogen peroxide (in 50 mM phosphate buffer at pH 7.0), and 150 µL of 5 mM KMnO_4_. Subsequently, the absorbance at 490 nm was measured. CAT enzyme was used as a reference standard, with concentrations ranging from 10 to 100 units/mL. The results were expressed as units/mg protein [32].

For the determination of superoxide dismutase (SOD) activity, the method described by Sun and colleagues was followed. A mixture solution of 0.5 mM xanthine (pH 7.4), 0.2 M phosphate buffer solution (KH_2_PO_4_) at pH 7.8, 0.01 M EDTA, and 15 µM cytochrome C (50:25:1:1 (v/v)) was prepared. The prepared sample (20 µL) was mixed with 200 µL of the mixture solution and 20 µL of xanthine oxidase (0.90 mU/mL). The absorbance was measured at 415 nm. SOD enzyme activity, ranging from 0 to 25 units/mL, was used as the reference standard. The results were expressed as units/mg protein [33].

The activity of glutathione peroxidase (GSH-Px) was assessed by mixing 20 µL of the prepared sample with a mixed solution containing 10 µL of 1 mM dithiothreitol (DTT), 10 mM monosodium phosphate (NaH_2_PO_4_) in distilled water, 1 mM sodium azide in 40 mM potassium phosphate buffer at pH 7.0, 10 µL of 50 mM glutathione solution, and 100 µL of 30% hydrogen peroxide. The reaction mixture was incubated at 25 °C for 10 min, followed by the addition of 10 µL of 10 mM DTNB. The absorbance at 412 nm was measured. GSH-Px enzyme activity, ranging from 1 to 5 units/mL, was used as the reference standard. The GSH-Px activity was expressed as units/mg protein [34].

To measure the level of malondialdehyde (MDA), an indicator of lipid peroxidation, the thiobarbituric acid reaction was employed. Briefly, 50 µL of the prepared sample was combined with a mixture solution consisting of 50 µL of 8.1% sodium dodecyl sulfate, 375 µL of 0.8% thiobarbituric acid, 375 µL of 20% acetic acid, and 150 µL of distilled water. The resulting mixture was then heated and maintained at 95 °C for 60 min. Subsequently, the reaction mixture was cooled using tap water and mixed with a solution comprising 1,250 µL of n-butanol and pyridine (at a ratio of 15:1) and 250 µL of distilled water. This mixture was subjected to centrifugation at 4,000 rpm for 10 min. The upper layer was collected, and the absorbance at 532 nm was measured. 1,1,3,3-tetramethoxypropane (TMP) at concentrations ranging from 0 to 15 µM, was used as a reference standard. The results were expressed as ng/mg protein [35].

### Western blotting analysis

To extract proteins from the SH-SY5Y cells, 1/5 (w/v) RIPA (radioimmunoprecipitation assay) buffer (Cell Signaling Technology, USA) was used. The buffer consisted of 20 mM Tris-HCl (pH 7.5), 150 mM NaCl, 1 mM Na_2_EDTA, 1 mM EGTA, 1% NP-40, 1% sodium deoxycholate, 2.5 mM sodium pyrophosphate, 1 mM beta-glycerophosphate, 1 mM Na_3_VO_4_, 1 µg/mL leupeptin, and 1 mM phenylmethanesulfonyl fluoride (PMSF) (Cell Signaling Technology, USA). The prepared sample was centrifuged at 12,000 × g at 4 °C for 10 min and the supernatant collected. The protein concentration was determined using a Thermo Scientific NanoDrop 2000c spectrophotometer (Thermo Fisher Scientific, Wilmington, Delaware, USA). Subsequently, 20 µg of cell lysate was mixed with Tris-Glycine SDS-PAGE loading buffer and heated at 95 °C for 10 min. Protein isolation was performed using sodium dodecyl sulfate-polyacrylamide gel electrophoresis (SDS-PAGE) by loading 20 µg of cell lysate onto an SDS-polyacrylamide gel. The separated protein bands were then transferred to a polyvinylidene fluoride (PVDF) membrane, washed with 0.05% TBS-T, and blocked with blocking buffer (5% skim milk in 0.1% TBS-T) at 25 °C for 1 h. The nitrocellulose membrane was subsequently incubated with the following antibodies at 4 °C overnight: anti-ERK1/2 (Cell Signaling Technology, USA; dilution 1:1,000), anti-CREB (Affinity Biosciences, UK; dilution 1:1,000), anti-Bcl-2  (Affinity Biosciences, USA; dilution 1:1,000), anti-caspase 3 (Cell Signaling Technology, USA; dilution 1:1,000), and anti-β-actin (Cell Signaling Technology, USA; dilution 1:2000). Following incubation, the membrane was washed with TBS-T (0.05%) and incubated with anti-rabbit IgG, HRP-linked antibody (Cell Signaling Technology, USA; dilution 1:2,000) at 25 °C for 1 h. The protein bands were visualized and quantified using the ECL detection system (GE Healthcare) and LAS-4000 luminescent image analyzer (GE Healthcare). ImageQuant TL v.7.0 image analysis software (GE Healthcare) was used to measure the band intensities for statistical analysis. The expression levels were normalized using anti-β-actin. The membranes were incubated and visualized with each antibody, followed by the internal control. Representative Western blot images were cropped to remove irrelevant portions and present only the proteins of interest, while the full-length blots are provided in the supplementary material. The data were presented as relative density compared to the naïve control group [36].

### Statistical analysis

The data is presented as the mean ± standard error of the mean (SEM). Statistical significance was evaluated using one-way analysis of variance (ANOVA), followed by the post hoc Tukey test. For comparisons between two groups, the student’s t-test was applied. A p-value of less than 0.05 was regarded as statistically significant. All statistical analyses were performed utilizing SPSS version 27 (IBM Corp., Released 2020, IBM SPSS Statistics for Mac OS).

## Results

### Bioactive compounds and their corresponding biological activities

The findings presented in Table 1 demonstrate that the aqueous extract of *Zea mays* L. *var. ceratina* contained active compounds, including total phenolic compounds, total flavonoids, and total anthocyanins, at concentrations of 121.13 ± 1.44 mg GAE/g of extract, 83.78 ± 2.94 mg quercetin/g of extract, and 29.67 ± 1.09 mg C3G/g of extract, respectively. The 50% hydroalcoholic extract boasts even higher levels of these bioactive compounds, with concentrations of 183.43 ± 2.12 mg GAE/g of extract, 118.17 ± 5.58 mg quercetin/g of extract, and 48.50 ± 3.75 mg C3G/g of extract. Similarly, the 95% hydroalcoholic extract also contained substantial levels of these bioactive compounds, with concentrations of 129.02 ± 1.94 mg GAE/g of extract, 90.18 ± 6.54 mg quercetin/g of extract, and 31.73 ± 2.41 mg C3G/g of extract.

**Table 1:** Main active compounds and primary anthocyanin derivatives of *Zea mays* L. *var. ceratina* extracts

Parameters	Units		Aqueous extract		50% hydro-alcoholic extract		95% hydro-alcoholic extract
**Active compounds**							
Total phenolic content	mg Gallic acid/g		121.13 ± 1.44		183.43 ± 2.12^aaa, ***^		129.02 ± 1.94
Total flavonoid content	mg Quercetin/g		83.78 ± 2.94		118.17 ± 5.58^aa, *^		90.18 ± 6.54
Total anthocyanins content	mg C3G /g		29.67 ± 1.09		48.50 ± 3.75^aa, *^		31.73 ± 2.41
**Primary anthocyanin derivatives**							
Cyanidin-3,5-diglucoside	µg /mg extract		-		6.75 ± 0.16		-
Cyanidin-3-glucoside	µg /mg extract		-		15.10 ± 0.01		-
Peonidin-3-glucoside	µg /mg extract		-		4.09 ± 0.03		-
Cyanidin-3-dimalonylglucoside	µg /mg extract		-		13.98 ± 1.20		-

Data are presented as mean ± SEM. ^aa, aaa ^*p* < 0.01 and 0.001, respectively; compared with aqueous extract, *, **, ***p < 0.05, 0.01 and 0.001, respectively; compared with 95% hydro-alcoholic extract

These results clearly demonstrate that the extraction of *Zea mays* L. *var. ceratina* using a 50% hydroalcoholic solution results in the highest concentration of total phenolic compounds, total flavonoids, and total anthocyanins. This difference is statistically significant (*p* < 0.001 for total phenolic compounds, *p* < 0.01 for total flavonoids, and *p* < 0.01 for total anthocyanins) when compared to the aqueous extract, and also significant (*p* < 0.001 for total phenolic compounds, *p* < 0.05 for total flavonoids, and *p* < 0.05 for total anthocyanins) when compared to the 95% hydroalcoholic extract.

**Fig. 1:**
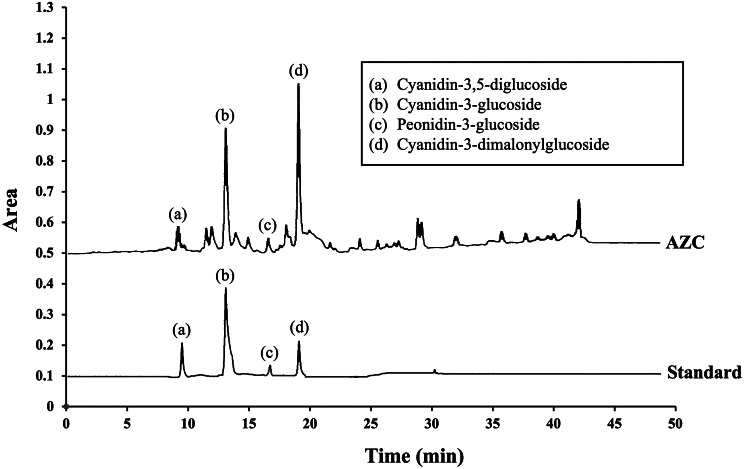
The chromatographic fingerprinting of primary anthocyanin derivatives of AZC. AZC: the anthocyanin-rich extract derived from *Zea mays* L. *var. ceratina*

Given the considerable potential demonstrated by the 50% hydroalcoholic extract, we opted to identify and quantify primary anthocyanin derivatives based on previous reports detailing major or primary anthocyanin derivatives [19, 37, 38]. The results, presented in Table 1; Fig. 1, reveal that each milligram of the 50% hydroalcoholic extract of *Zea mays* L. *var. ceratina* contains concentrations of 6.75 ± 0.16 µg/mg of cyanidin-3,5-diglucoside, 15.10 ± 0.01 µg/mg of cyanidin-3-glucoside, 4.09 ± 0.03 µg/mg of peonidin-3-glucoside, and 13.98 ± 1.20 µg/mg of cyanidin-3-dimalonylglucoside. These findings suggest that the primary anthocyanin derivative prevalent in the 50% hydroalcoholic extract of *Zea mays* L. *var. ceratina* is cyanidin-3-glucoside, which is consistent with previous findings [19, 37, 38].

To validate the beneficial effects of the anthocyanin-rich substance derived from the 50% hydroalcoholic extract, we assessed its biological activities relevant to dementia, including antioxidant and anti-inflammatory effects. The results in Table 2 reveal that the 50% hydroalcoholic extract exhibited significantly lower EC_50_ values in antioxidant assays (DPPH, FRAP, and ABTs) (*p* < 0.001, 0.01, and 0.05, respectively) compared to the aqueous extract, as well as lower EC_50_ values (*p* < 0.001, 0.01, and 0.05, respectively) compared to the 95% hydroalcoholic extractions. Moreover, the 50% hydroalcoholic extract displayed the lowest EC_50_ values for COX-2 suppression activity, although no significant difference was observed among the various extracts. Consequently, the 50% hydroalcoholic solution was selected for the preparation of the anthocyanin-rich extract from *Zea mays* L. *var. ceratina* (AZC) for further investigative experiments.

**Table 2:** Biological activities of various extracts of *Zea mays* L. *var. ceratina*

Parameters	Units	Aqueous extract	50% hydro-alcoholic extract	95% hydro-alcoholic extract	Standard reference
**Antioxidant activities**					
DPPH	EC_50_ (µg/mL)	76.28 ± 5.46	33.41 ± 0.57^aaa, ***^	68.99 ± 3.57	43.33 ± 0.02, Trolox
FRAP	EC_50_ (µg/mL)	56.24 ± 4.34	24.18 ± 2.55^aa, **^	54.72 ± 2.50	30.32 ± 0.01, Trolox
ABTS	EC_50_ (µg/mL)	52.62 ± 0.32	39.96 ± 0.05^a, *^	48.35 ± 0.18	41.73 ± 0.02, Trolox
**Inflammatory marker**					
COX-II	EC_50_ (µg/mL)	117.67 ± 2.13	98.72 ± 5.06^a^	104.69 ± 4.58	32.47 ± 0.01, Indomethacin

Data are presented as mean ± SEM. ^a, aa, aaa ^*p* < 0.05, 0,01 ,and 0.001, respectively; compared with aqueous extract, ^*, **, *** ^*p* < 0.05, 0,01 ,and 0.001, respectively; compared with 95% hydro-alcoholic extract

### Cytotoxicity of hydrogen peroxide and AZC on the viability of SH-SY5Y cells

The study findings revealed that treatment of SH-SY5Y cells with hydrogen peroxide had deleterious effects, including decreased cell viability, induced apoptosis, and impaired scavenging enzyme activity [30]. To establish a cytotoxic model using hydrogen peroxide, SH-SY5Y cells were exposed to various concentrations (25, 50, 100, 200, 400, and 800 µM) for 24 h, and cell viability was assessed using the MTT assay. Based on our prior publication [30], we determined that the optimal concentration for subsequent experiments was 200 µM of hydrogen peroxide treatment for a duration of 24 h.

**Fig. 2:**
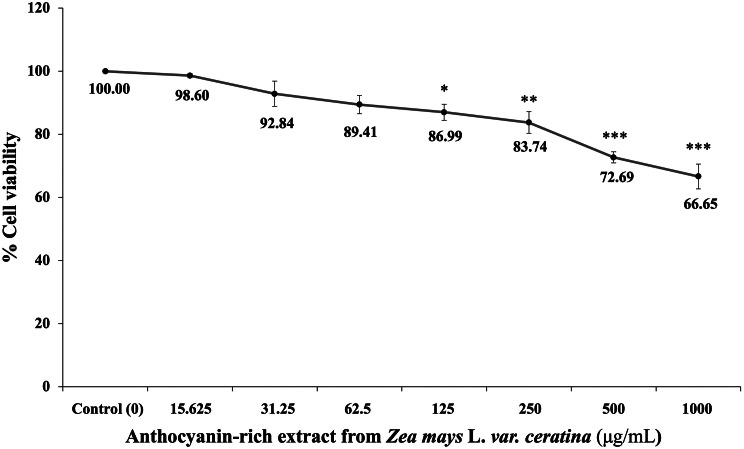
Effect of the AZC on the viability of SH-SY5Y cells. Data are presented as mean ± SEM. ^*, **, ***^p < 0.05, 0.01 and 0.001, respectively; compared with naïve control. AZC: the anthocyanin-rich extract derived from *Zea mays* L. *var. ceratina*

To assess the cytotoxicity of AZC, SH-SY5Y cells were exposed to various concentrations of extract (15.625, 31.25, 62.5, 125, 250, 500, and 1,000 µg/mL) for 24 h, and the results are depicted in Fig. 2. The data indicated a decrease in cell viability with increasing concentration: 98.60 ± 0.64, 92.84 ± 4.03, 89.41 ± 2.86, 86.99 ± 2.54, 83.74 ± 3.45, 72.69 ± 1.77, and 66.65 ± 3.91, respectively. Notably, significant reductions in cell viability were observed at concentrations of 125, 250, 500, and 1,000 µg/mL (*p* < 0.05, 0.01, 0.001, and 0.001, respectively; compared to the control group). Consequently, the doses of 31.25 and 62.5 µg/mL were identified as the maximum non-toxic doses of AZC for SH-SY5Y cells. These doses were chosen for further investigation to determine the protective effect of AZC against hydrogen peroxide-induced cytotoxicity.

### Effects of AZC on the viability of SH-SY5Y cells

**Fig. 3:**
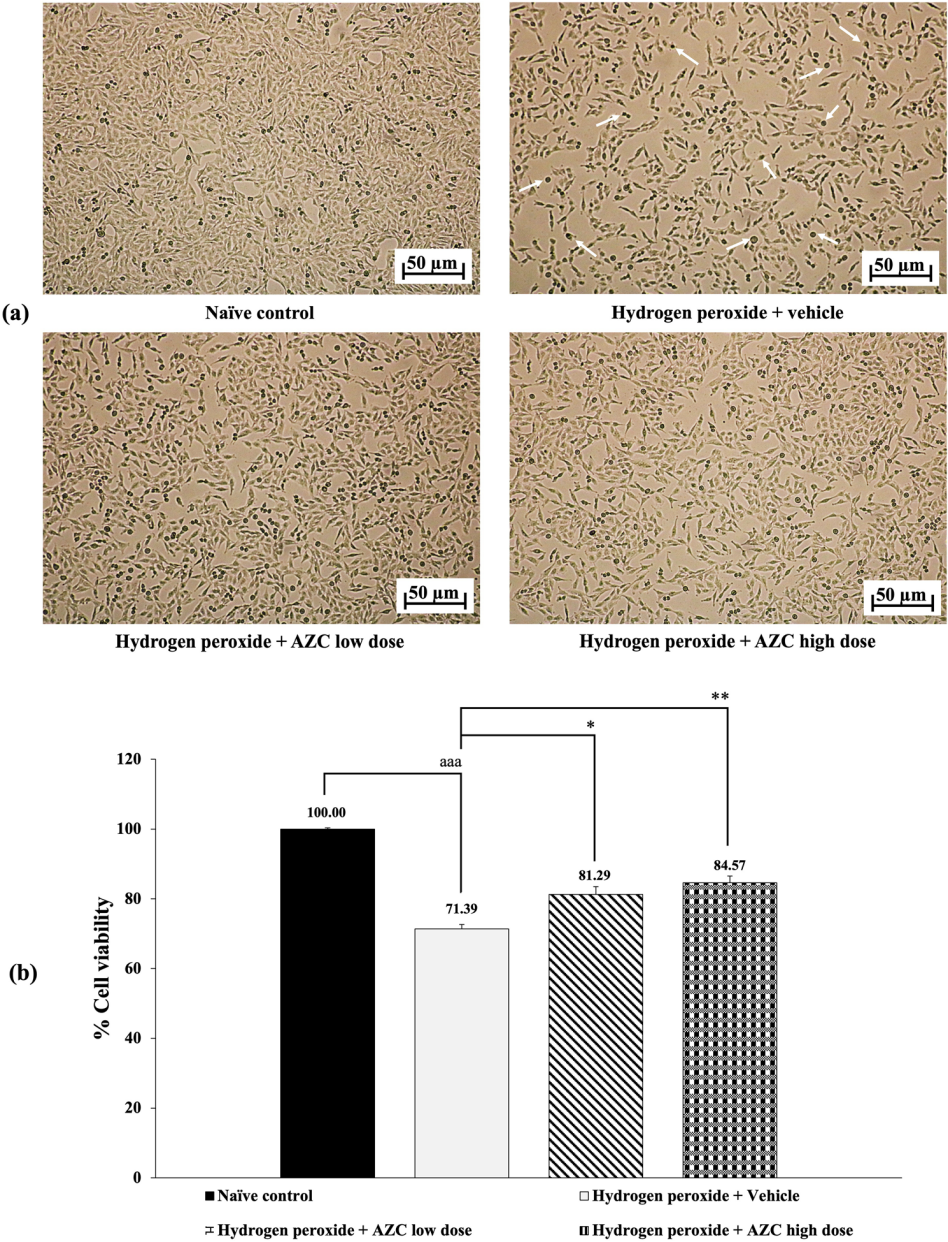
Effect of AZC on hydrogen peroxide induced cytotoxicity in SH-SY5Y cells. (a) Light microscopy of SH-SY5Y cell morphology at 40x magnification. (b) % cell viability of SH-SY5Y cells. Data are presented as mean ± SEM. ^aaa^*p* < 0.001; compared to naïve control, ^*, **^p < 0.05 and 0.01, respectively; compared to SH-SY5Y cells treated with hydrogen peroxide, and which had received the vehicle. hydrogen peroxide: hydrogen peroxide at dose of 200 µM; AZC low dose and AZC high dose: the anthocyanin-rich extract derived from *Zea mays* L. *var. ceratina* at doses of 31.25 and 62.5 µg/mL, respectively

Figure 3 illustrates the neuroprotective effect of AZC. The data demonstrates that when SH-SY5Y cells were exposed to hydrogen peroxide and received the vehicle, a significant reduction in cell viability was observed (*p* < 0.001 compared to the naïve control group). Additionally, the cell morphology displayed shorter processes and adopted a round shape, as indicated by the white arrows in Fig. 3(a). In contrast, treatment with AZC at concentrations of 31.25 and 62.5 µg/mL effectively countered this decrease in cell viability (*p* < 0.05 and 0.01, respectively, when compared to the group treated with hydrogen peroxide + vehicle).

### Effects of AZC on intracellular ROS production

**Fig. 4:**
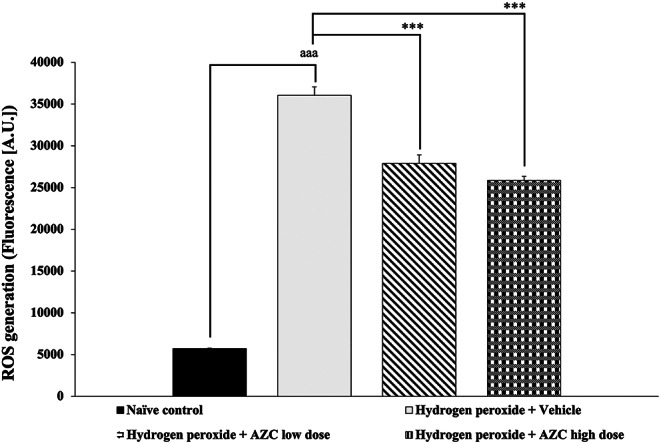
Effect of AZC on reactive oxygen species generation in SH-SY5Y cell toxicity induced by hydrogen peroxide. Data are presented as mean ± SEM. ^aaa^*p* < 0.001; compared to naïve control, ^***^p < 0.001; compared to SH-SY5Y cells treated with hydrogen peroxide, and which had received the vehicle. hydrogen peroxide: hydrogen peroxide at dose of 200 µM; AZC low dose and AZC high dose: the anthocyanin-rich extract derived from *Zea mays* L. *var. ceratina* at doses of 31.25 and 62.5 µg/mL, respectively

Given the crucial role of oxidative stress in the development of various neurodegenerative disorders, including dementia such as Alzheimer’s disease, Parkinson’s disease, and vascular dementia, elevated levels of reactive oxygen species (ROS) can contribute to neuronal damage and cognitive decline [39]. Our study aimed to investigate the impact of AZC on ROS production in SH-SY5Y cells exposed to hydrogen peroxide-induced cytotoxicity. The findings, as depicted in Fig. 4, indicate a significant increase in ROS production in SH-SY5Y cells treated with hydrogen peroxide and the vehicle (*p* < 0.001; compared to the naïve control group). Interestingly, all doses of AZC treatment effectively alleviated the rise in ROS production (*p* < 0.001 for all; compared to the group treated with hydrogen peroxide + vehicle).

### Effects of AZC on the oxidative stress status

The protective effects of AZC on oxidative stress status, including MDA levels and the levels of key scavenger enzymes (CAT, SOD, and GSH-Px), are presented in Table 3. It was observed that SH-SY5Y cells treated with hydrogen peroxide and receiving the vehicle displayed a significant increase in MDA levels (*p* < 0.001; compared to the naïve control group) and a decrease in CAT, SOD, and GSH-Px levels (*p* < 0.05, 0.01, and 0.01, respectively; compared to the naïve control group).

**Table 3:** The effect of the AZC on oxidative stress markers in SH-SY5Y cell toxicity induced by hydrogen peroxide

Treatment groups	MDA (ng/mg protein)	CAT (units/mg protein)	SOD (units/mg protein)	GSH-Px (units/mg protein)
Naïve control	11.44 ± 1.22	28.88 ± 1.75	41.77 ± 0.77	25.41 ± 1.28
Hydrogen peroxide + Vehicle	27.70 ± 2.27^aaa^	15.73 ± 1.74^a^	25.70 ± 0.36^aa^	16.81 ± 0.49^aa^
Hydrogen peroxide + AZC low dose	16.74 ± 0.11^**^	29.61 ± 2.15^*^	30.15 ± 1.69	22.02 ± 0.85^*^
Hydrogen peroxide + AZC high dose	16.71 ± 0.68^**^	27.71 ± 3.15^*^	35.55 ± 2.03^*^	21.27 ± 0.82^*^

Data are presented as mean ± SEM. ^a, aa, aaa^*p* < 0.05, 0.01 and 0.001, respectively; compared to naïve control, ^*,**^p < 0.05 and 0.01, respectively; compared to SH-SY5Y cells which received hydrogen peroxide and vehicle. AZC low dose and AZC high dose : the anthocyanin-rich extract obtained from *Zea mays* L. *var. ceratina* at doses of 31.25 and 62.5 µg/mL, respectively

However, SH-SY5Y cells treated with hydrogen peroxide and AZC at a dose of 31.25 µg/mL exhibited a significant decrease in MDA levels, accompanied by an increase in CAT and GSH-Px levels (*p* < 0.01, 0.05, and 0.05, respectively; compared to the group treated with hydrogen peroxide + vehicle). Nevertheless, no significant change in SOD levels was observed in SH-SY5Y cells treated with hydrogen peroxide and AZC at a dose of 31.25 µg/mL.

Similarly, treatment with AZC at a dose of 62.5 µg/mL resulted in a significant decrease in MDA levels (*p* < 0.01; compared to the group treated with hydrogen peroxide + vehicle) and a significant increase in all scavenging enzymes, including CAT, SOD, and GSH-Px (*p* < 0.05 for all; compared to the group treated with hydrogen peroxide + vehicle).

### Effects of AZC on the alteration of ERK1/2 signal transduction

**Fig. 5:**
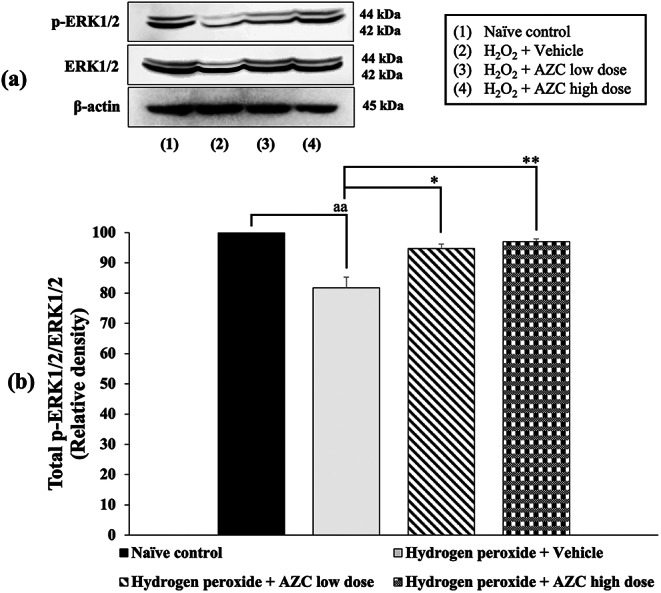
Effect of AZC on the expression of ERK1/2 in the hydrogen peroxide induced SH-SY5Y cell toxicity was detected by Western blotting. (a) Representative Western blot showing the levels of total p-ERK1/2/ERK1/2. (b) Relative density of total p-ERK1/2/ERK1/2. Data are presented as mean ± SEM. ^aa^*p* < 0.01; compared to naïve control, ^*, **^p < 0.05 and 0.01, respectively; compared to SH-SY5Y cells treated with hydrogen peroxide, and which had received the vehicle. ERK1/2: extracellular signal-related kinases 1 and 2; H_2_O_2_: hydrogen peroxide at dose of 200 µM; AZC low dose and AZC high dose: the anthocyanin-rich extract derived from *Zea mays* L. *var. ceratina* at doses of 62.5 and 125 µg/mL, respectively

Considering the pivotal role of ERK1/2 signal transduction in neuronal cell death, we investigated the impact of AZC on ERK1/2 phosphorylation in hydrogen peroxide-induced SH-SY5Y cytotoxicity, and the results are presented in Fig. 5. The data indicate that SH-SY5Y cells treated with hydrogen peroxide and the vehicle exhibited a significant decrease in the expression of ERK1/2 phosphorylation (*p* < 0.01; compared to the naïve control group). However, all doses of AZC treatment effectively reversed the decrease in ERK1/2 phosphorylation induced by hydrogen peroxide (*p* < 0.05 and 0.01, respectively; compared to the hydrogen peroxide + vehicle group).

### Effects of AZC on the alteration of CREB

**Fig. 6:**
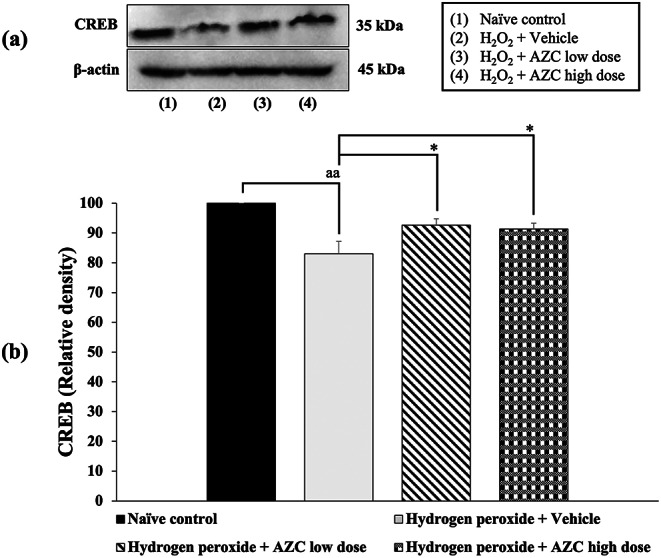
Effect of AZC on the expression of CERB in the hydrogen peroxide induced SH-SY5Y cell toxicity was detected by Western blotting. (a) Representative Western blot showing the levels of total CREB. (b) Relative density of total CREB. Data are presented as mean ± SEM. ^aa^*p*< 0.01; compared to naïve control, ^*^p < 0.05; compared to SH-SY5Y cells treated with hydrogen peroxide, and which had received the vehicle. CREB: cAMP response element-binding protein; H_2_O_2_: hydrogen peroxide at dose of 200 µM; AZC low dose and AZC high dose: the anthocyanin-rich extract derived from *Zea mays* L. *var. ceratina* at doses of 62.5 and 125 µg/mL, respectively

To confirm the neuroprotective effects of AZC, we examined CREB, a transcription factor crucial for neuroprotection. The results are depicted in Fig. 6. SH-SY5Y cells treated with hydrogen peroxide and receiving the vehicle exhibited a significant decrease in CREB expression (*p* < 0.01; compared to the naïve control group). Interestingly, AZC treatment at doses of 31.25 and 62.5 µg/mL effectively mitigated the reduction in CREB expression induced by hydrogen peroxide (*p* < 0.05 for both; compared to the hydrogen peroxide + vehicle group).

### Effects of AZC on markers of apoptosis

**Fig. 7:**
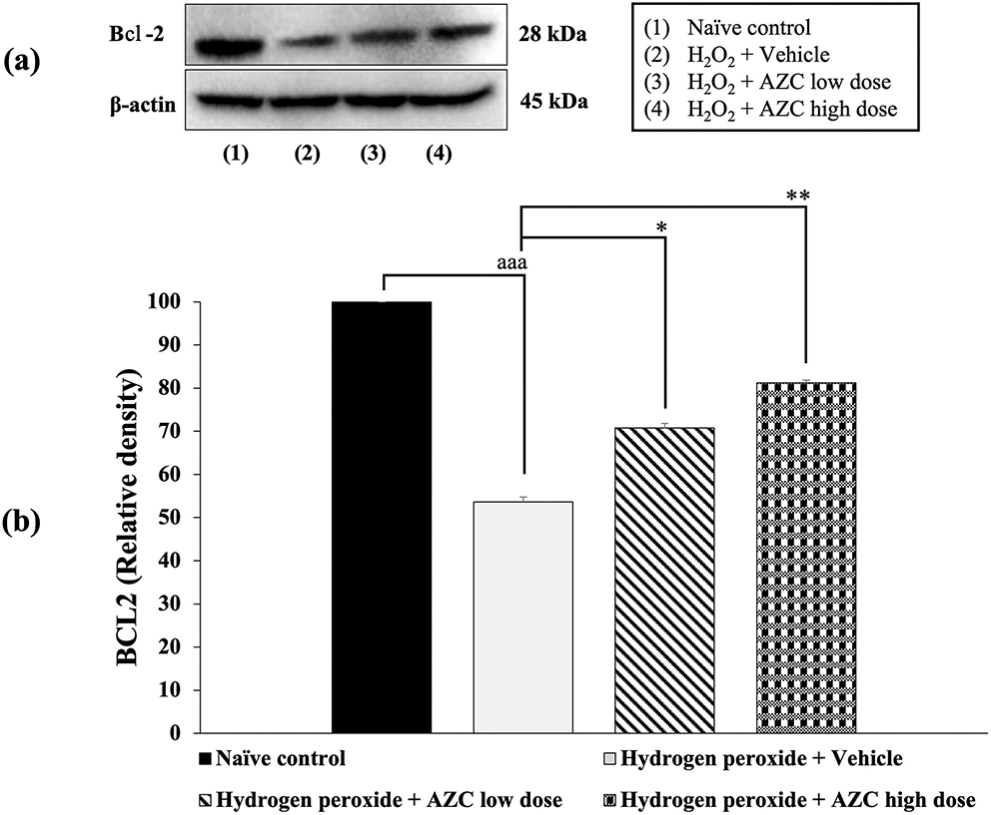
Effect of AZC on the expression of Bcl-2  in the hydrogen peroxide induced SH-SY5Y cell toxicity was detected by Western blotting. (a) Representative Western blot showing the levels of total Bcl-2 . (b) Relative density of total Bcl-2 . Data are presented as mean ± SEM. ^aaa^*p* < 0.001; compared to naïve control, ^*, **^p < 0.05 and 0.01, respectively; compared to SH-SY5Y cells treated with hydrogen peroxide, and which had received the vehicle. Bcl-2 : B-cell lymphoma 2; H_2_O_2_: hydrogen peroxide at dose of 200 µM; AZC low dose and AZC high dose: the anthocyanin-rich extract derived from *Zea mays* L. *var. ceratina* at doses of 62.5 and 125 µg/mL, respectively

**Fig. 8:**
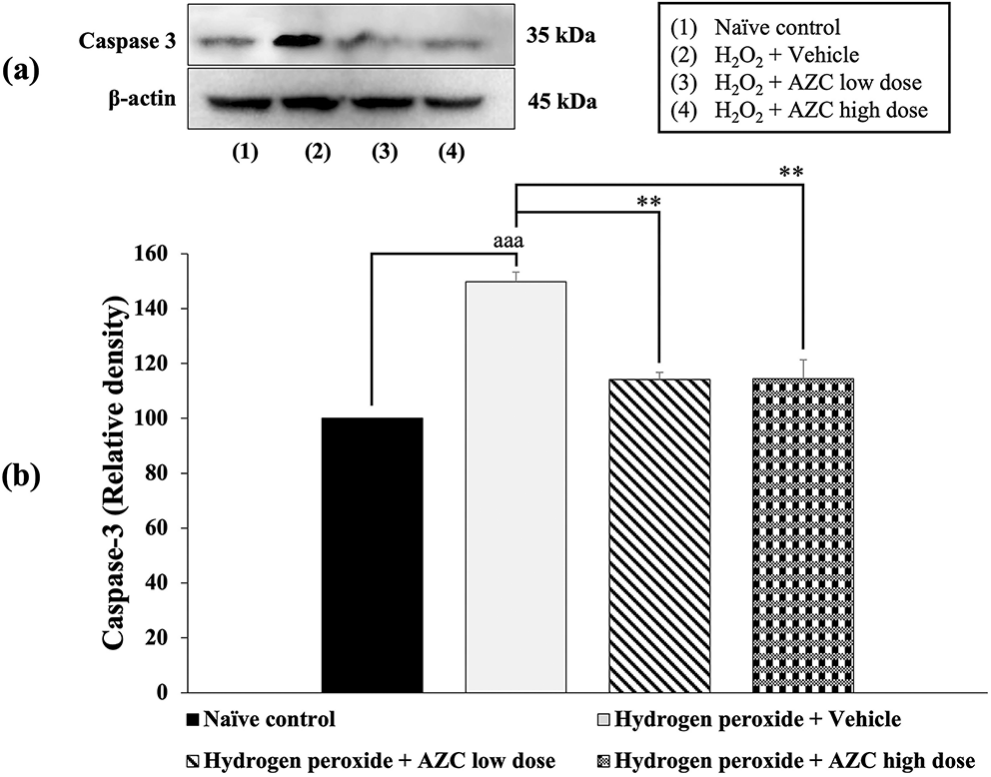
Effect of AZC on the expression of caspase 3 in the hydrogen peroxide induced SH-SY5Y cell toxicity was detected by Western blotting. (a) Representative Western blot showing the levels of total caspase 3. (b) Relative density of total caspase 3. Data are presented as mean ± SEM. ^aaa^*p* < 0.001; compared to naïve control, ^**^p< 0.01; compared to SH-SY5Y cells treated with hydrogen peroxide, and which had received the vehicle. H_2_O_2_: hydrogen peroxide at dose of 200 µM; AZC low dose and AZC high dose: the anthocyanin-rich extract derived from *Zea mays* L. *var. ceratina* at doses of 62.5 and 125 µg/mL, respectively

Undesired apoptosis performs a role in various conditions, including neurodegenerative diseases. To investigate how AZC exerts its neuroprotective effects, we examined the levels of Bcl-2 , a marker of anti-apoptotic inhibition, and caspase 3, a marker of apoptotic induction. The results shown in Figs. 7 and 8 indicate that SH-SY5Y cells treated with hydrogen peroxide and the vehicle experienced a significant reduction in Bcl-2  expression and an increase in caspase 3 expression (*p* < 0.001 for both; compared to the naïve control group).

Importantly, all doses of AZC treatment effectively reversed the decrease in Bcl-2 expression (*p* < 0.05 for 31.25 µg/mL, and *p* < 0.01 for 62.5 µg/mL; compared to the group treated with hydrogen peroxide + vehicle). Furthermore, all AZC treatments also significantly suppressed the expression of caspase 3 (*p* < 0.05 for 31.25 µg/mL, and *p* < 0.01 for 62.5 µg/mL; compared to the group treated with hydrogen peroxide + vehicle).

## Discussion

Anthocyanins, natural pigments belonging to the flavonoid family, are widely recognized for their presence in various fruits, vegetables, and plants, contributing to their distinct colors. In recent years, they have gained significant attention for their potential health benefits, particularly in the field of neuroprotection [14, 15, 40]. Several studies have explored anthocyanins’ neuroprotective properties, yielding promising results. These compounds possess potent antioxidant capabilities, enabling them to neutralize harmful free radicals and ameliorate oxidative stress within neuronal cells [41, 42]. Additionally, anti-inflammatory effects of anthocyanins have been demonstrated through inhibition of inflammatory pathways and by reducing the production of pro-inflammatory molecules [16]. Both oxidative stress and inflammation are common features of various neurological conditions and can lead to neuronal cell death and cognitive decline [43]. Through their ability to reduce oxidative stress and inflammation, anthocyanins may help protect against neurodegenerative processes.

Our findings align with prior studies in the literature, as we have observed that the 50% hydroalcoholic extract of *Zea mays* L. *var. ceratina* contained a significant quantity of phenolics and flavonoids, particularly anthocyanins and their derivatives. Furthermore, this extract exhibited remarkably low EC_50_ values for antioxidant activities, including DPPH, FRAP, and ABTS. Additionally, it demonstrated noteworthy suppressive effects on COX-2. These discoveries provide further substantiation for the existing body of evidence regarding the advantageous properties of anthocyanins.

The current results have demonstrated the cytoprotective effect of AZC against hydrogen peroxide-induced SH-SY5Y cytotoxicity. Additionally, reductions in ROS production, MDA level, and caspase 3 expression were observed, accompanied by increased scavenging enzymes such as CAT, SOD, and GSH-Px, along with the upregulation of ERK1/2, CREB, and Bcl-2  expression.

Hydrogen peroxide is widely recognized as a toxic agent that induces oxidative stress, generating highly reactive hydroxyl radicals that inflict damage to cells. Several studies have indicated that hydrogen peroxide-induced oxidative stress can activate signaling pathways involved in apoptotic cell death [44, 45]. The interplay between oxidative stress and apoptosis in the context of dementia is complex. Oxidative stress can trigger apoptosis, and in turn, apoptosis can exacerbate oxidative stress by releasing reactive oxygen species and disrupting antioxidant defense mechanisms [46]. Excessive oxidative stress can also disrupt the balance between pro-apoptotic and anti-apoptotic factors, leading to heightened apoptotic signaling and neuronal cell death [47].

Our results validate that hydrogen peroxide treatment increases oxidative stress by reducing the activities of catalase (CAT), superoxide dismutase (SOD), and glutathione peroxidase (GSH-Px), while elevating malondialdehyde (MDA), an indicator of oxidative stress, and ROS levels. However, treatment with AZC effectively reversed these changes, suggesting that it may enhance the activities of scavenger enzymes and reduce MDA and ROS levels. The reduction in oxidative stress, as evidenced by the decrease in MDA and ROS, contributed to the mitigation of neuronal cell injury and the promotion of neural cell survival. These findings agree with previous studies documented in the literature.

Furthermore, our findings demonstrate that hydrogen peroxide treatment induced apoptosis, as substantiated by elevated caspase 3 levels and diminished Bcl-2 levels [30, 48]. These outcomes are consistent with previous investigations indicating that oxidative stress influences undesirable apoptotic mechanisms, ultimately leading to cell death [49]. Considering these findings, we propose that AZC’s positive modulation effect on SH-SY5Y cell viability may be partly attributed to its ability to improve oxidative stress, resulting in the downregulation of caspase 3 expression and the upregulation of Bcl-2 expression. Consequently, AZC suppresses undesired apoptosis and promotes increased cell viability.

It has been extensively documented that the ERK1/2-CREB- Bcl-2 pathway plays a pivotal role in cell survival and neuroprotection [50, 51]. This signaling cascade entails the activation of extracellular signal-regulated kinases 1 and 2 (ERK1/2), which subsequently activate the transcription factor known as cAMP response element-binding protein (CREB). Upon activation, CREB initiates the expression of various target genes, including Bcl-2, a protein well-known for its anti-apoptotic properties [52].

ERK1/2 can be stimulated by a range of extracellular factors, including therapeutic agents and phytochemical substances. Once activated, ERK1/2 translocates to the nucleus, where it phosphorylates and activates CREB [53]. The activation of CREB, a transcription factor with the ability to bind to specific DNA sequences and regulate the transcription of target genes, leads to the increased expression of Bcl-2  [54]. Dysregulation of the ERK1/2-CREB- Bcl-2 pathway has been implicated in numerous neurodegenerative conditions, such as dementia [55]. Diminished pathway activation can render neurons more vulnerable to stress, impair cell survival, and heighten apoptosis, thereby contributing to the progression of neurodegeneration.

Our data substantiate these existing findings. The results emphasize that AZC treatment may exert a neuroprotective influence by activating the ERK1/2 signaling pathway, which subsequently triggers CREB activation and the consequent upregulation of Bcl-2 expression. Consequently, apoptosis is suppressed, leading to an enhancement in neuronal cell viability.

Collectively considering all the data, this study presents compelling evidence of the neuroprotective properties of AZC against hydrogen peroxide-induced cytotoxicity in SH-SY5Y cells. The potential mechanism underlying this effect involves the mitigation of undesired apoptotic processes and improvement of the oxidative stress status, ultimately resulting in enhanced cell viability.

## Conclusion

**Fig. 9:**
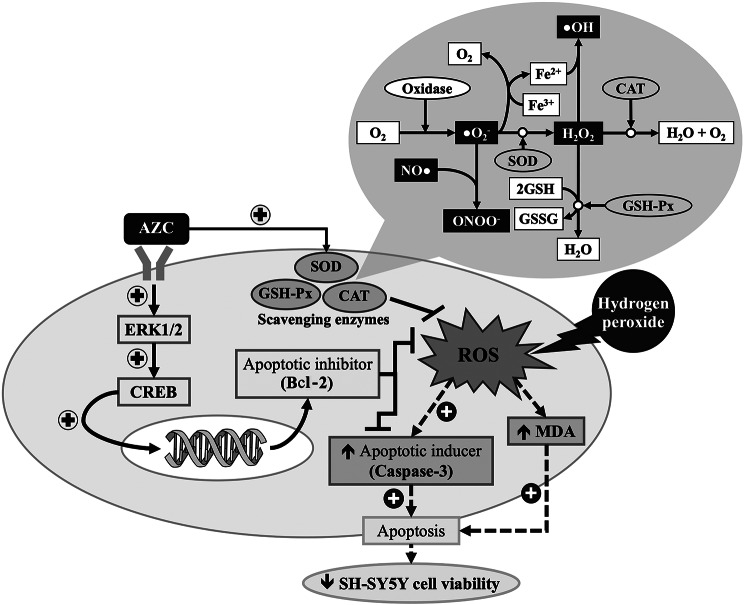
The schematic diagram demonstrated the neuroprotective effect of AZC in the hydrogen peroxide induced SH-SY5Y cytotoxicity. AZC: the anthocyanin-rich extract derived from *Zea mays* L. *var. ceratina*; ERK1/2: extracellular signal-related kinases 1 and 2; Bcl-2: B cell lymphoma 2; CREB: cAMP response element-binding protein; MDA: malondialdehyde; SOD: superoxide dismutase; CAT: catalase; GSH-Px: glutathione peroxidase; ROS: reactive oxygen species

This study represents the first investigation demonstrating the neuroprotective effects of AZC. The underlying mechanisms potentially involve multitarget actions, including the alleviation of oxidative stress and the suppression of excessive apoptosis mediated through the ERK1/2-CREB- Bcl-2 pathway, as illustrated in Fig. 9. Consequently, AZC exhibits substantial potential as a functional ingredient for the development of neuroprotective agents, particularly in the context of neurodegenerative disorders such as dementia. However, toxicity studies are imperative to ensure the safety of AZC consumption before proceeding to in vivo and clinical investigations.

### Electronic supplementary material

Below is the link to the electronic supplementary material.

Supplementary Material 1

## Data Availability

The datasets generated and/or analyzed during the current study are available upon reasonable request from the corresponding author.
