# Supplementary material for: An anthocyanin-rich extract from Zea mays L. var. ceratina alleviates neuronal cell death caused by hydrogen peroxide-induced cytotoxicity in SH-SY5Y cells
Source: BMC Complement Med Ther. 2024 Apr 17;24:162. doi: 10.1186/s12906-024-04458-6 (PMC11025150; doi:10.1186/s12906-024-04458-6)
Supplement: Supplementary file 1 — Supplementary Material 1 [file 12906_2024_4458_MOESM1_ESM.pdf]

Original Blot of p-ERK1/2 Expression

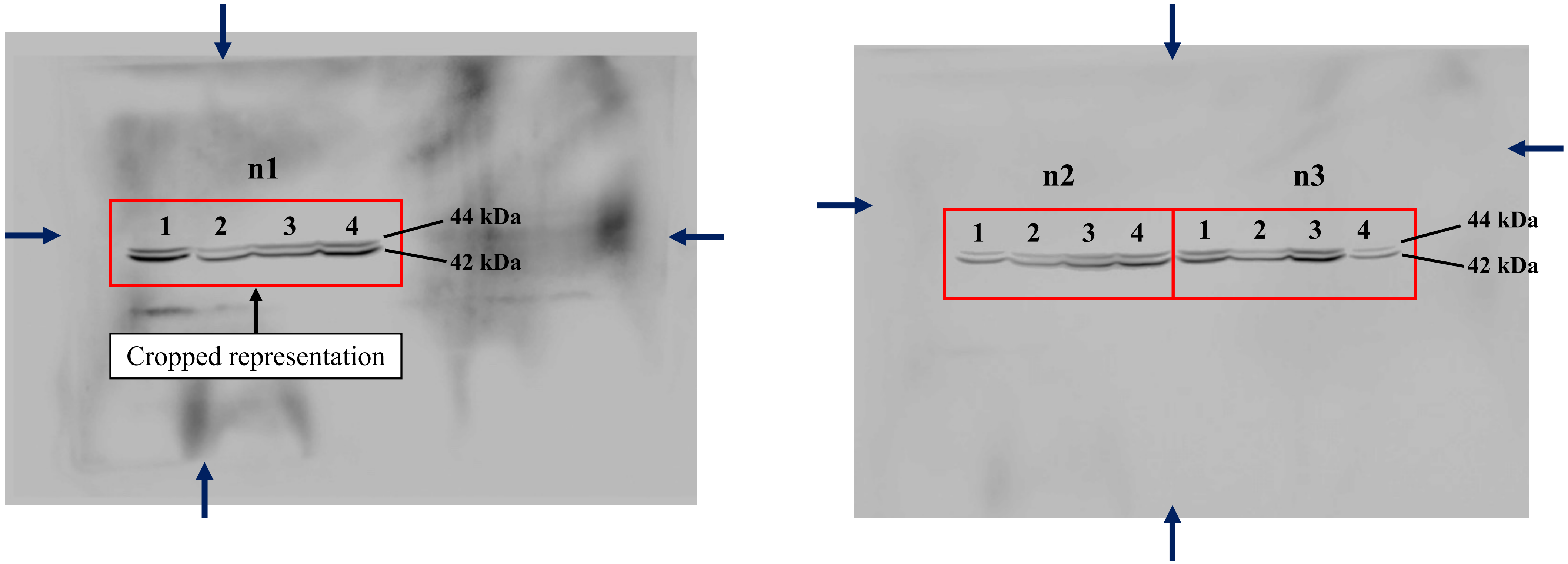

Supplementary Figure 1: Western blotting was used to detect the expression of p-ERK1/2 in SH-SY5Y cells induced by hydrogen peroxide toxicity. The groups included (1) naïve control, (2) H_2O_2 + vehicle, (3) H_2O_2 + AZC low dose, and (4) H_2O_2 + AZC high dose. The full-length membranes are shown, with membrane edges indicated by the dark blue arrows.

Original Blot of ERK1/2 Expression

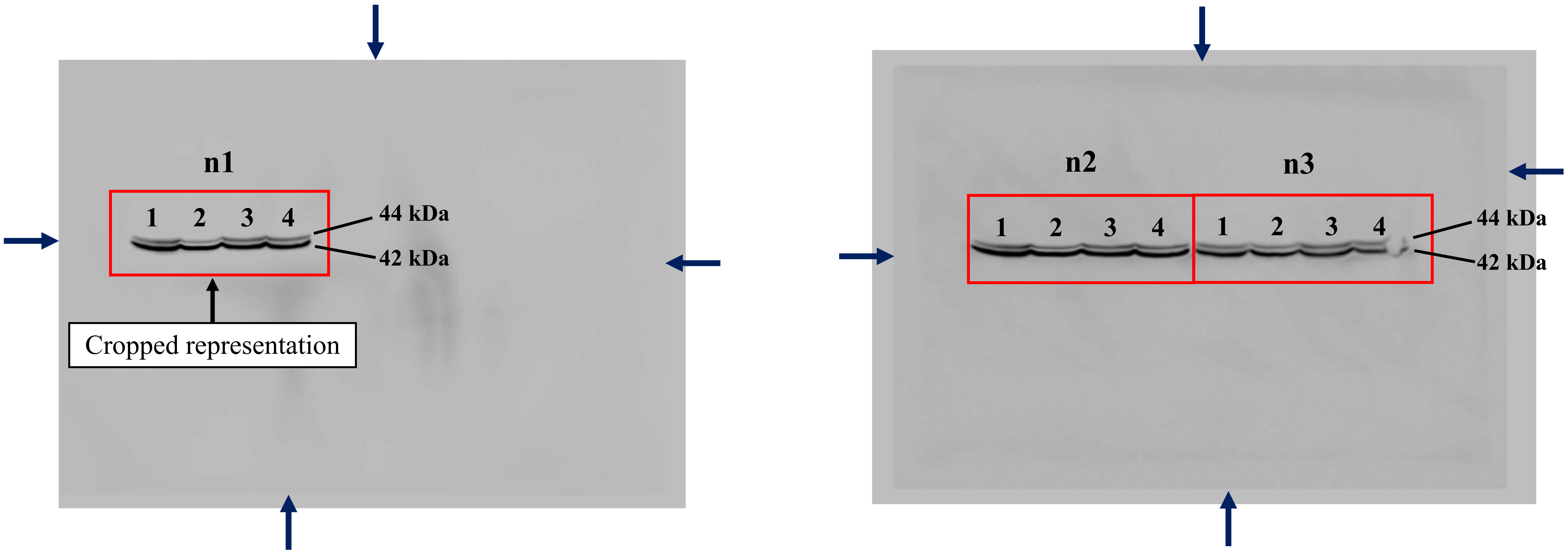

Supplementary Figure 2: Western blotting was used to detect the expression of ERK1/2 in SH-SY5Y cells induced by hydrogen peroxide toxicity. The groups included (1) naïve control, (2) H_2O_2 + vehicle, (3) H_2O_2 + AZC low dose, and (4) H_2O_2 + AZC high dose. The full-length membranes are shown, with membrane edges indicated by the dark blue arrows.

Original Blot of CREB Expression

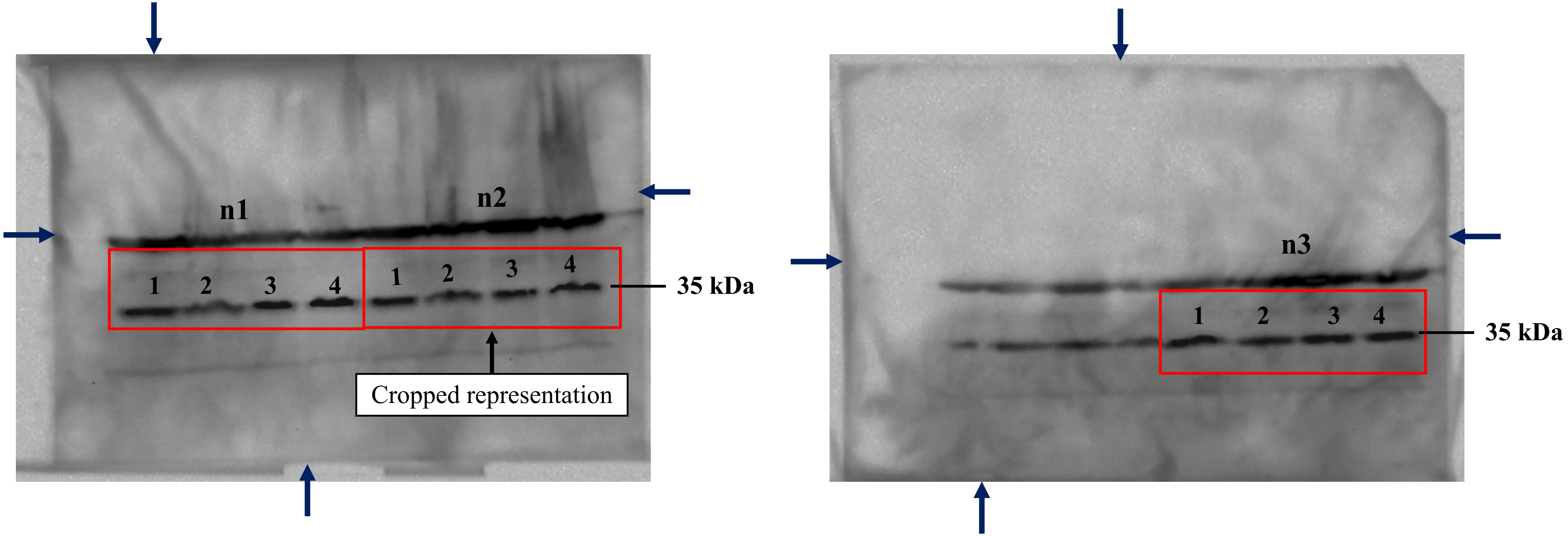

Supplementary Figure 3: Western blotting was used to detect the expression of CREB in SH-SY5Y cells induced by hydrogen peroxide toxicity. The groups included (1) naïve control, (2) H_2O_2 + vehicle, (3) H_2O_2 + AZC low dose, and (4) H_2O_2 + AZC high dose. The full-length membranes are shown, with membrane edges indicated by the dark blue arrows.

Original Blot of BCL-2 Expression

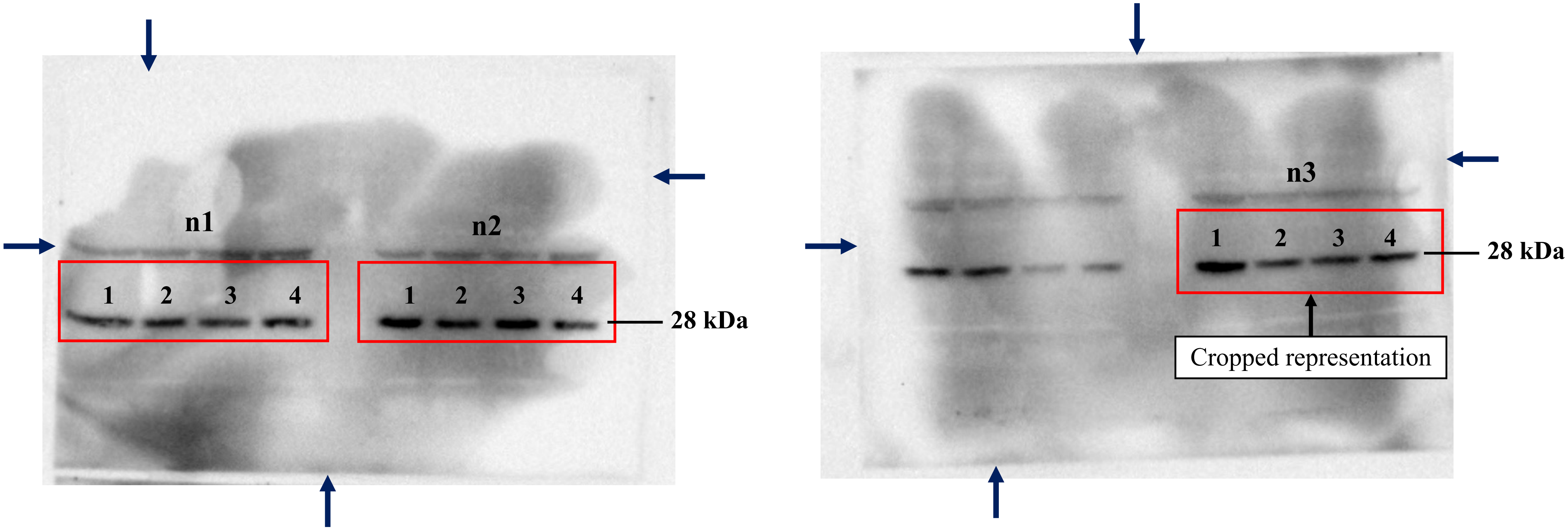

Supplementary Figure 4: Western blotting was used to detect the expression of BCL-2 in SH-SY5Y cells induced by hydrogen peroxide toxicity. The groups included (1) naïve control, (2) H_2O_2 + vehicle, (3) H_2O_2 + AZC low dose, and (4) H_2O_2 + AZC high dose. The full-length membranes are shown, with membrane edges indicated by the dark blue arrows.

Original Blot of Caspase-3 Expression

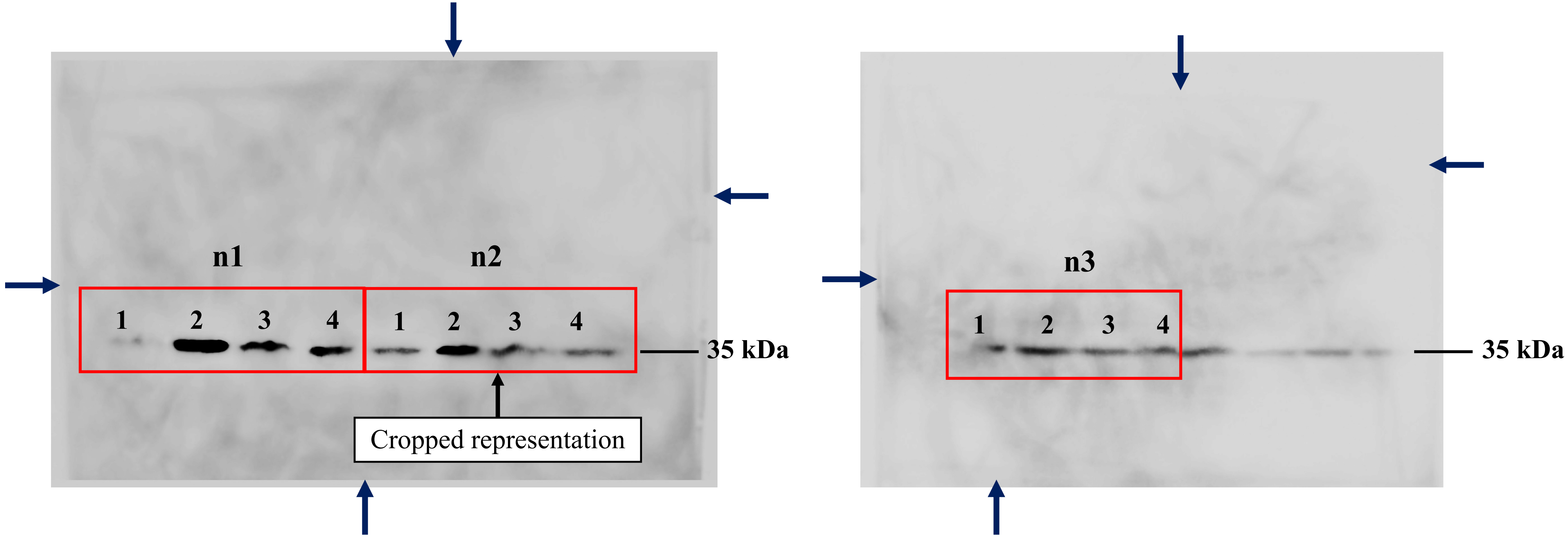

Supplementary Figure 5: Western blotting was used to detect the expression of Caspase-3 in SH-SY5Y cells induced by hydrogen peroxide toxicity. The groups included (1) naïve control, (2) H_2O_2 + vehicle, (3) H_2O_2 + AZC low dose, and (4) H_2O_2 + AZC high dose. The full-length membranes are shown, with membrane edges indicated by the dark blue arrows.
